# A (cholesterol) crystal clear path to inflammasome activation in atherosclerosis

**DOI:** 10.1016/j.jlr.2024.100554

**Published:** 2024-05-03

**Authors:** Xiang Li, Tamer Sallam

**Affiliations:** 1 Division of Cardiology, Department of Medicine, UCLA, Los Angeles, CA, USA; 2 Department of Physiology, UCLA, Los Angeles, CA, USA; 3 Molecular Biology Institute, UCLA, Los Angeles, CA, USA

Inflammation is strongly linked to atherosclerosis. Immune cells are highly enriched in atherosclerotic lesions and elevated inflammatory markers independently predict cardiovascular events (1). Based on data from the LoDoCo-2 and COLCOT clinical trials, the Food and Drug Administration for the first time approved the use of the anti-inflammatory drug colchicine for reducing atherosclerotic cardiovascular disease (2, 3). The NLR family pyrin domain–containing 3 (NLRP3) inflammasome is often nominated as another anti-inflammatory target in atherosclerosis. NLRP3 is an intracellular “danger” sensor that regulates innate immune cell responses and release of proinflammatory cytokines such as interleukin 1 beta and interleukin 18 (4). Studies have shown that the NLRP3 inflammasome is activated in cholesterol overload states and plays a direct role in promoting atherosclerosis (5).

The mechanism by which cholesterol leads to inflammasome activation is not well defined. It has been proposed that accumulation of cholesterol crystals through phagolysosomal mechanisms leads to NLRP3 activation (5). Cellular cholesterol, however, is mostly partitioned and regulated in other compartments (6). The majority of cholesterol is present in the plasma membrane, where three distinct pools exist: *1*) an accessible pool that is highly dynamic and signals to the endoplasmic reticulum (ER) to dictate homeostatic cholesterol regulation, *2*) a sphingomyelin-sequestered pool that can be recruited with sphingomyelin hydrolysis, and *3*) an essential pool that cannot be depleted without altering cellular architecture and morphology (6). In this issue of the journal Yalcinkaya *et al.* (7) suggest that transport of cholesterol between the plasma membrane cholesterol and ER instigates inflammasome activation (Fig. 1). Under lipid loading conditions, treatment of macrophages with ALOD-4, which blocks accessible plasma membrane to ER cholesterol transport, reduced inflammasome-driven interleukin 1 beta secretion. Reciprocally, lipopolysaccharides-primed macrophages treated with acetylated LDL and sphingomyelinase, which liberates membrane cholesterol for movement to the ER, showed increased IL-1B and inflammasome activation markers. These studies are in line with other work showing, in a Niemann-Pick disease, type C1 deletion model, that ER cholesterol trafficking impacts NLRP3 activity (8). To reinforce the idea that plasma membrane to ER cholesterol content can drive inflammasome activation, the authors use an RNA interference approach targeting a key transporter of cholesterol movement. The Aster family of proteins mediates nonvesicular cholesterol transport from plasma membrane to ER (9). Knockdown of Aster-B reduced IL-1 secretion and caspase-1 cleavage in the context of macrophage lipid overload. Importantly, these perturbations had no effect on toll-like receptor 4 signaling. Collectively, these studies suggest that cholesterol movement to the ER acts as a key checkpoint for inflammasome activation.

**Fig. 1:**
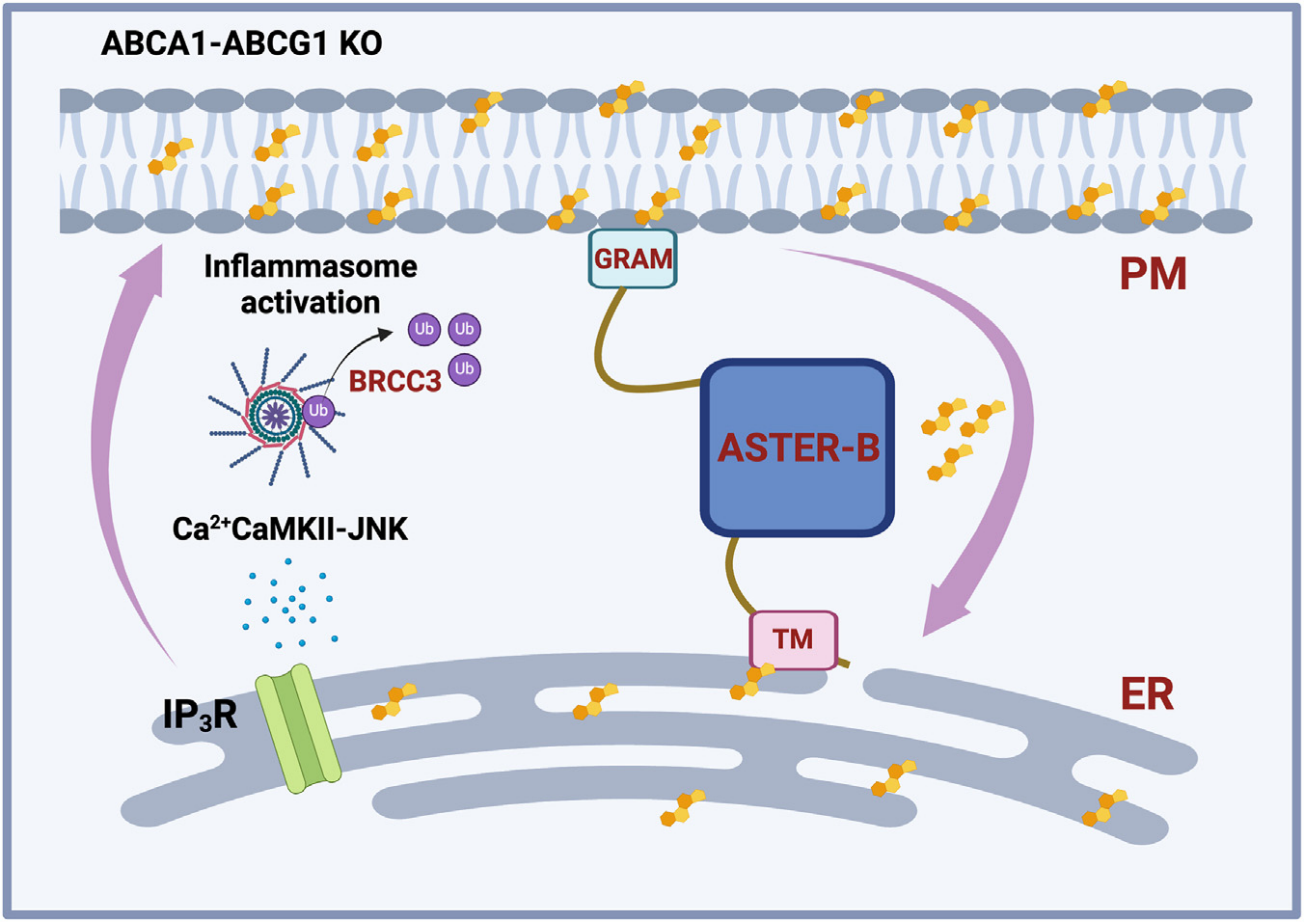
Proposed mechanism of how plasma membrane (PM) to endoplasmic reticulum (ER) cholesterol transport leads to NLRP3 inflammasome. Lipid-loaded Abca1-Abcg1–deficient macrophages transport cholesterol from the PM to the ER via Aster-B. An increase in ER cholesterol stimulates the inositol triphosphate-3 receptor (IP_3_R) and calcium release. A calcium-dependent CaMKII/JNK axis induces NLRP3 deubiquitylation by BRCC3 and inflammatory cytokine release. BRCC3, BRCA1/BRCA2-containing complex subunit 3; CaMKII, Ca^2+^/calmodulin-dependent protein kinase II; GRAM, GRAM domain of Aster; JNK, c-Jun N-terminal kinase, NLRP3, NLR family pyrin domain–containing 3; TM, transmembrane domain of Aster; Ub, ubiquitin.

How does a change in ER cholesterol content lead to inflammasome activation? Using a combination of pharmacologic, genetic, and cell biologic approaches, the authors go on to show that an ER cholesterol-calcium switch induces a change in NLRP3 deubiquitylation via Ca ^2+^/calmodulin-dependent protein kinase II/c-Jun N-terminal kinase. NLRP3 is known to be critically regulated by phosphorylation c-Jun N-terminal kinase and deubiquitylation (BRCA1/BRCA2-containing complex subunit 3 [BRCC3]) (10). Upon cholesterol accumulation, NLRP3–BRCC3 interaction is enhanced resulting in inflammasome complex assembly and activation. In an atherosclerosis model, pharmacologic inhibition of BRCC3 or genetic deletion of the BRCC3 cofactor ABRO1 in immune cells reduced lesion area, inflammasome activation markers, and NETosis.

The findings reported by Yalcinkaya and colleagues are significant for the number of reasons. First, they provide evidence that posttranslational modification of inflammasome components can dictate crucial cardiometabolic traits such as atherosclerosis development. Second, the work expands the modes by which NLRP3 inflammasome couples metabolic and immune signaling. The overarching concept that cholesterol movement dictates inflammasome activation consolidates well with known functions of the ER as a cholesterol sensor and site of excess cholesterol esterification. This proposes the ER as a rheostat that simultaneously regulates cholesterol and inflammasome activation. Much like cholesterol movement, the localization of inflammasome components changes dynamically with cellular cues and complex activation (4). It is therefore conceivable that the escort activity of ER resident proteins could be an important driver of inflammasome activation in addition to cholesterol movement. Recent work showed that NLRP3 complexes with SREBF chaperone and that translocation of SCAP-NLRP3 to Golgi are required for maximal activation (11). Future work should be able to disentangle the contributions of cholesterol regulation, ER chaperone activity, and organelle architectural changes in inflammasome assembly and activation.

Contextualizing the results of the study by Yalcinkaya *et al.* is important. Most of the perturbations disrupting cholesterol movement were done in a myeloid-deficient ABCA1-ABCG1 mouse model, suggesting that extreme cholesterol overload is needed for the proposed mechanisms to become operational. These conditions are in line with work from the group of investigators showing that “multiple hits,” such those occurring with clonal hematopoiesis of indeterminate potential mutations or ABC transporter deletion, may be needed for the inflammasome to meaningfully impact atherosclerosis (12). These observations hint that targeting the inflammasome for cardiovascular risk mitigation may only be useful in select contexts.

Based on this work, it is tempting to speculate that Aster-B deletion in immune cells under conditions of cholesterol excess reduces inflammasome activation and atherosclerosis. Loss of Aster is associated with reduced ER cholesterol and oxysterol production, which in turn could lead to a reduction in liver X receptor activation (9). In a chronic atherosclerosis model, loss of Aster-B could have important effects on membrane rigidity that could impact lesion development through different mechanisms. Thus, the effects of Aster-B in immune cells on atherosclerosis may not be straight forward. Finally, recent work highlights a role for Aster proteins in promoting plasma membrane to ER cholesterol movement during physiologic conditions, such as fasting, reverse cholesterol transport, and gut cholesterol absorption (13, 14). Indeed, cholesterol movement to the ER is thought to occur routinely in the absence of extreme lipid loading or priming, which hints that there may be other checkpoints or collaborative factors that inform inflammasome activation in response to cholesterol. We eagerly await additional insights into inflammasome mechanisms in atherosclerosis.

## Conflict of interest

The authors declare that they have no conflicts of interest with the contents of this article.
